# Rare case of intracranial hemorrhage associated with seoul virus infection diagnosed by metagenomic next‐generation sequencing

**DOI:** 10.1002/jcla.23616

**Published:** 2020-10-21

**Authors:** Dan Xie, Wen Xu, Ying Xian, Xiaofeng Yuan, Zhenchao Huang, Jingya You, Xiaogang Bi

**Affiliations:** ^1^ Department of General Intensive Care Unit Lingnan Hospital The Third Affiliated Hospital Sun Yat‐Sen University Guangzhou China; ^2^ Department of Neurosurgery Lingnan Hospital The Third Affiliated Hospital Sun Yat‐Sen University Guangzhou China

**Keywords:** intracranial hemorrhage, mNGS, seoul virus

## Abstract

**Background:**

Seoul virus (SEOV) is a Hantavirus and the causative pathogen of Hemorrhagic Fever with Renal Syndrome (HFRS). Diagnosing SEOV infection is difficult because the clinical presentations are often undistinguishable from other viral or bacterial infections. In addition, diagnostic tools including serological and molecular assays are not readily available in the clinical settings.

**Case Report:**

A 57‐year‐old male presented with fever and a sudden loss of consciousness in November 2019. Computed tomography (CT) scan showed subdural hematoma, subfalcine herniation, and brain infarction. He developed thrombocytopenia and elevated transaminases, but no rashes or obvious kidney damage. He reported having a rat bite. HFRS was suspected. The Hantavirus IgG was positive, and the metagenomic next‐generation sequencing (mNGS) detected SEOV sequences directly in the blood.

**Conclusion:**

This report highlights the importance of suspecting SEOV infection in febrile patients with thrombocytopenia and elevated liver enzymes despite the absence of hemorrhagic manifestations of skin and renal syndromes. Next‐generation sequencing is a powerful tool for pathogen detection. Intracranial hemorrhage and brain infarction as extrarenal manifestations of HFRS are rare but possible as demonstrated in this case.

## Graphical abstract

Intracranial hemorrhage and brain infarction as extrarenal manifestations of HFRS are rare. mNGS can be a powerful tool to detect SEOV directly in the blood. Clinicians should be aware of various extrarenal manifestations of SEOV infections.

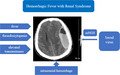

## 1 BACKGROUND

Seoul virus (SEOV) is a member of Hantavirus family, which has three segments of negative‐sense, single‐stranded RNA genomes.^1^ The small (S) segment encodes a nucleoprotein, the medium (M) segment encodes 2 membrane glycoproteins (Gn and Gc), and the large (L) segment encodes an RNA‐dependent RNA polymerase. SEOV infection is a worldwide public health threat in humans. The virus, harbored by Rattus norvegicus and R.rattus rats, is one of major causative etiologies of Hemorrhagic Fever with Renal Syndrome (HFRS) in China.^2^ Clinically, the typical HFRS manifestations include five consecutive phases: febrile, hypotensive shock, oliguric, polyuric, and convalescent.^3^ The general clinical features include fever, myalgias, hemorrhage, thrombocytopenia, leukocytosis, renal failure, and shock in most severe cases. Elevation of liver enzymes is characteristic of HFRS caused by SEOV.^4^ But these presentations are non‐specific for HFRS, which are often undistinguishable from other viral or bacterial infections. The serological assays to detect Hantavirus IgM and IgG offered by the state or regional Centers for Disease Control and Prevention (CDC) can confirm HFRS, but are not specific to SEOV. Molecular assays to detect SEOV viral RNA is also not available in most hospitals. Metagenomic next‐generation sequencing (mNGS) is a powerful tool for the detection and identification of pathogens directly from the specimen. In this report, we describe a highly unusual case of SEOV infection diagnosed by mNGS in a patient with intracranial hemorrhage but without typical hemorrhagic manifestations of skin or renal syndromes.

## 2 CASE REPORT

**FIGURE 1:**
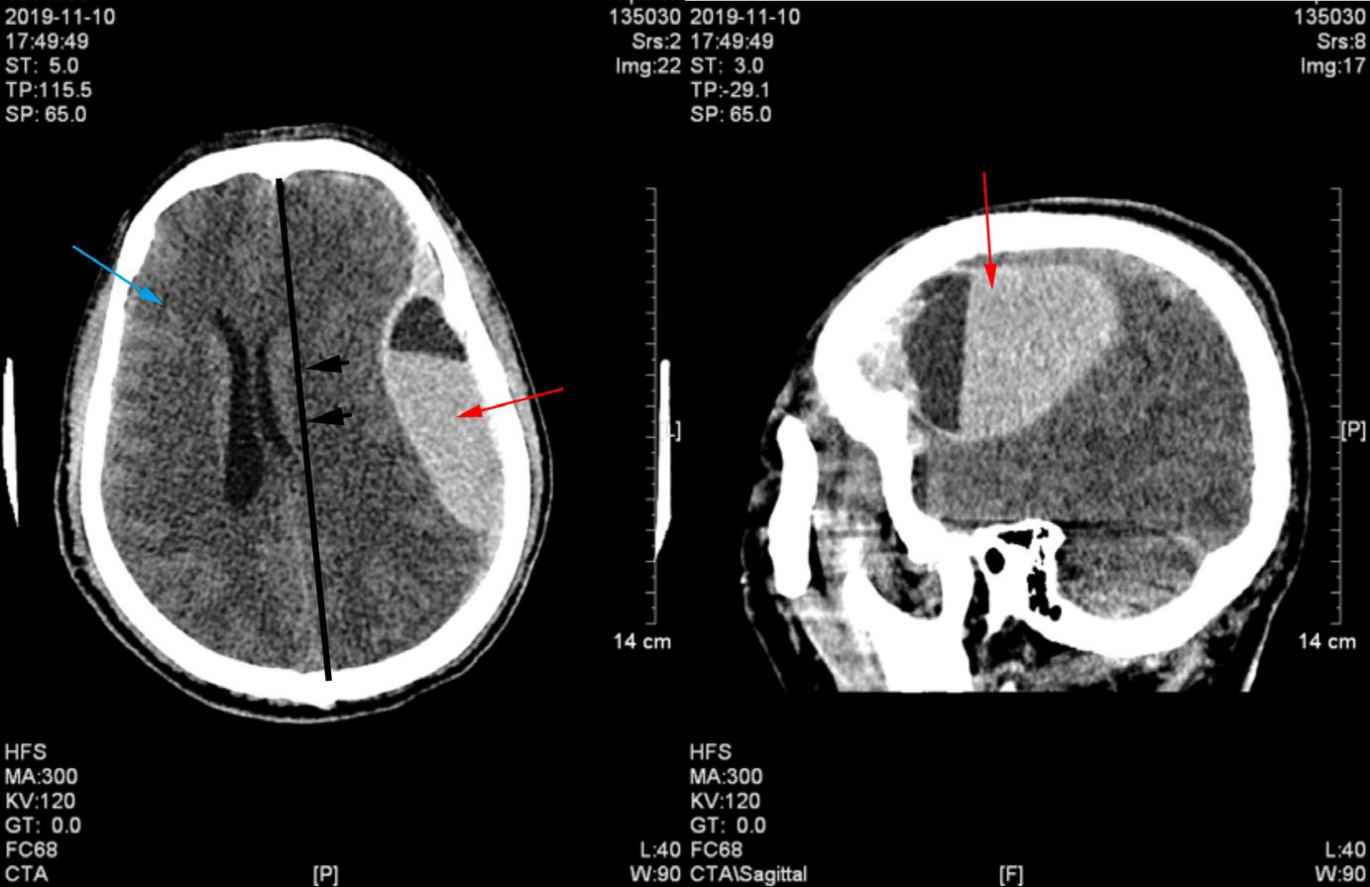
Head CT on admission indicated subdural hematoma (red arrow), brain infarction (blue arrow), and subfalcine herniation (dark arrow)

**FIGURE 2:**
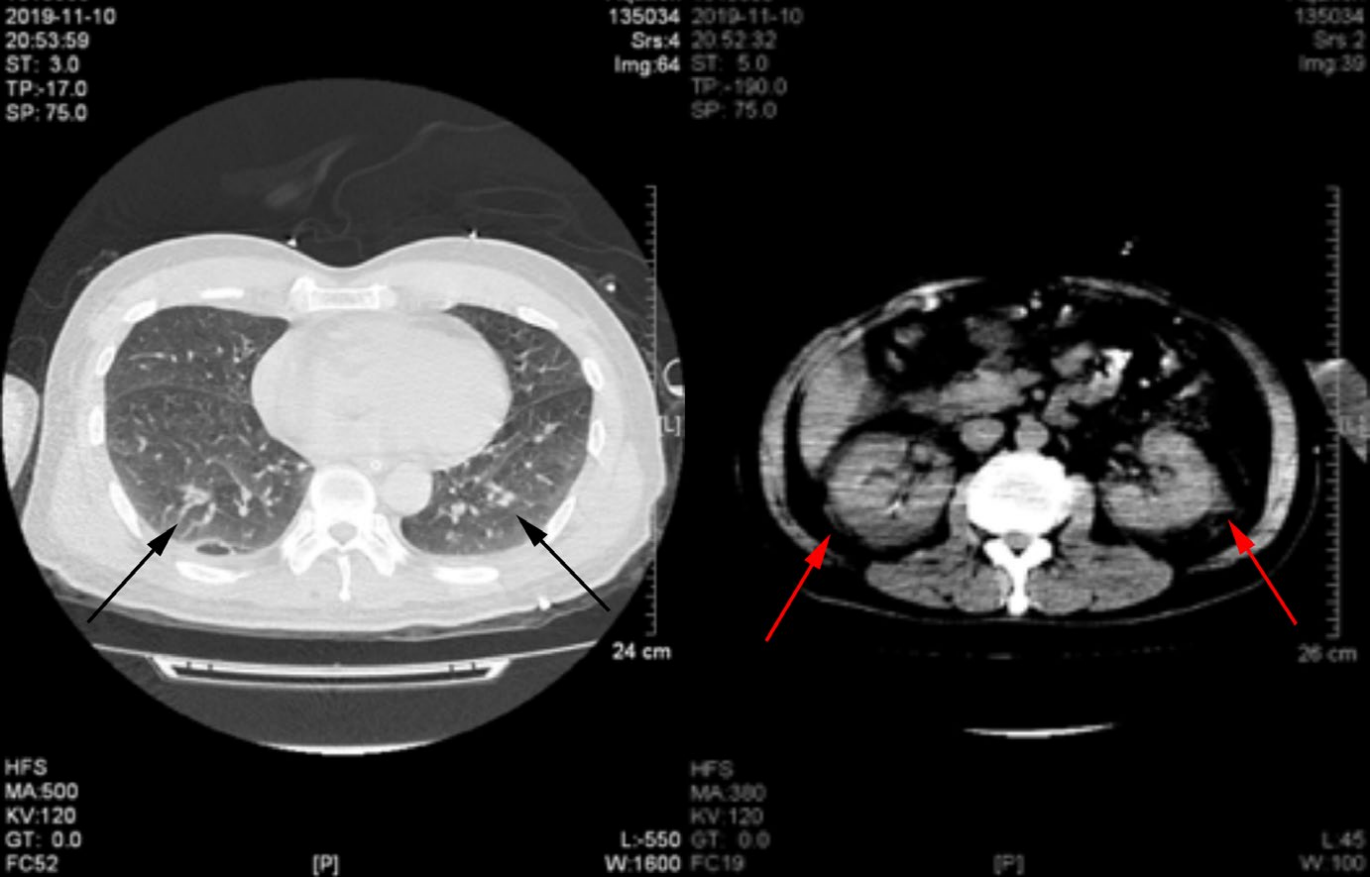
The chest and kidney CT on ICU admission, which suggested slight bilateral inflammation (left panel, black arrow) of lower lungs and perirenal inflammation (right panel, red arrow)

**FIGURE 3:**
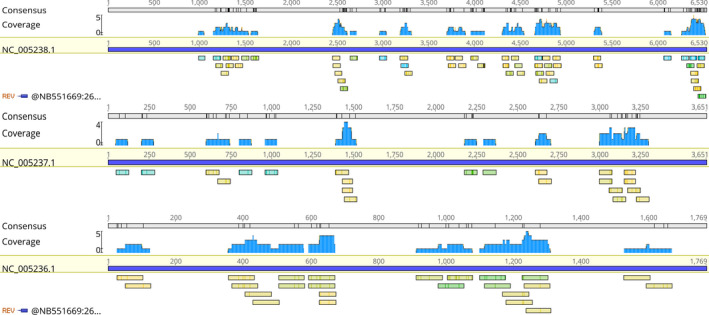
SEOV sequences detected by mNGS and mapped to an SEOV reference genome (S segment: NC_005236.1, M segment: NC_005237.1, L segment: NC_005238.1)

**FIGURE 4:**
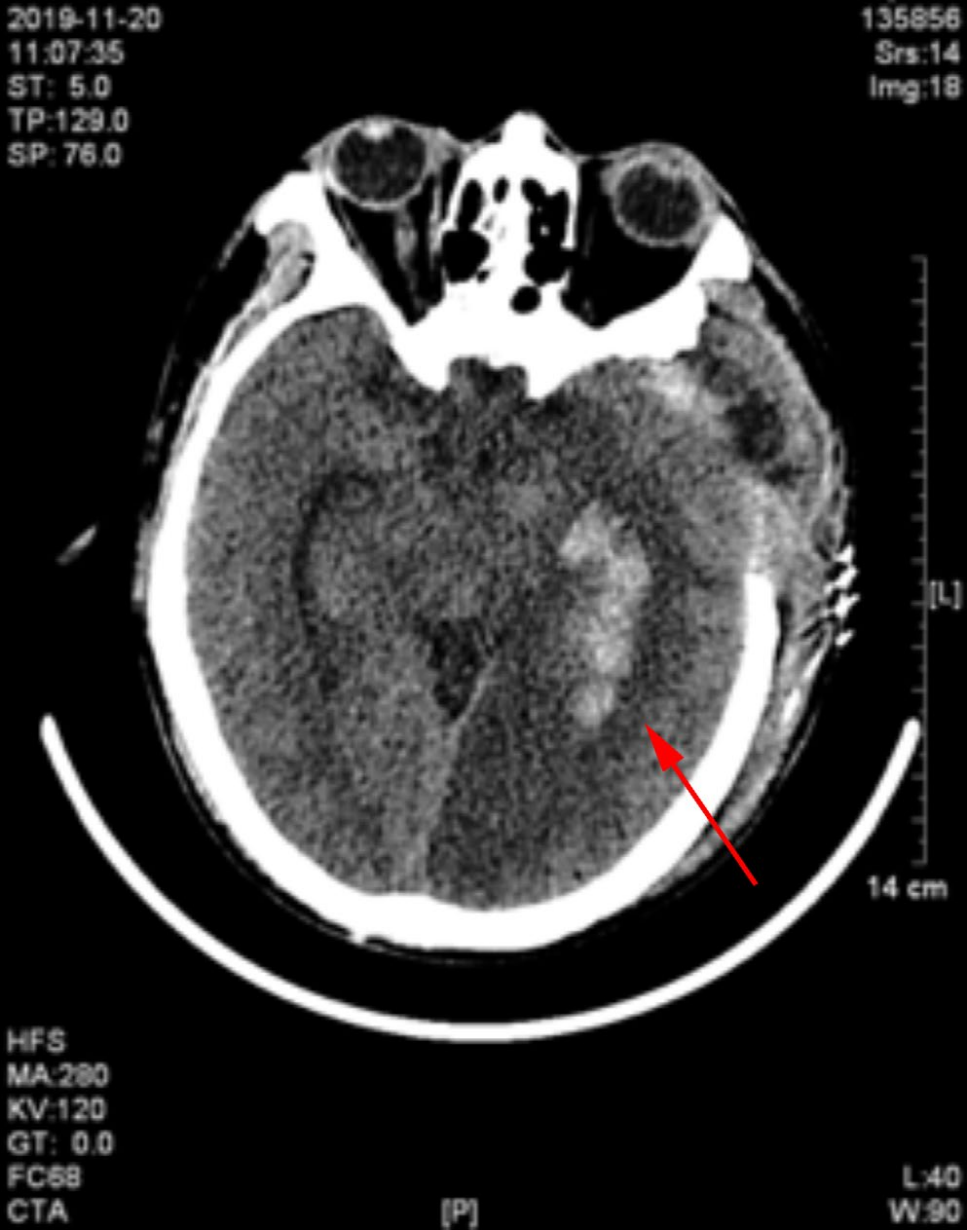
The Head CT revealed new intracranial hemorrhage on ICU day 10 (red arrow)

A 57‐year‐old previously healthy male was transferred to our hospital in November 2019 after a sudden loss of consciousness. Four days prior, the patient developed fever (maximum temperature 39°C) of unknown origin. He was admitted to a local hospital and was diagnosed with Dengue fever after the Dengue virus IgM was tested weakly positive. During the hospitalization, he suddenly lost consciousness and started vomiting. He had no significant medical history (particularly no anti‐platelet or anticoagulant history), no recent travel history, and no history of cerebral trauma. Physical examination showed blood pressure of 161/82 mmHg, heart rate of 97 beats/minutes, respiratory rate of 25/minutes, and temperature of 37.8℃. He was in a coma and did not response to voice. The Glasgow Coma Scale (GCS) score was 6 points. The diameter of his left and right pupil was 3.0mm and 4.0mm, respectively. The direct and indirect light reflex was slow. Limb movement was observed, and his chest, cardiac, and abdominal examination was normal. There were no rashes, petechia, ecchymoses, or other skin lesions. Blood tests performed 1 day before admission at the local hospital showed a white blood cell (WBC) count of 8.32 × 10E^9^/L, hemoglobin (Hb) level of 171 g/L, hematocrit of 52.5%, platelet count of 31 × 10E^9^/L, aspartate aminotransferase (AST) of 272 IU/L, alanine aminotransferase (ALT) of 225 IU/L, blood urea nitrogen (BUN) of 6.47 mmol/L, and serum creatinine of 83.4 µmol/L. Computed tomography scan of head indicated left subdural hemorrhage, subfalcine hernia, and brain infarction (Figure 1), there was no abnormality in the CT angiography (CTA). CT scan of whole abdomen suggested perirenal inflammation and chest CT showed slight bilateral inflammation of lower lungs (Figure 2). Surgical removal of subdural hematoma and decompressive craniectomy were performed immediately despite the low platelet count (20 × 10E^9^/L). He was then transferred to the intensive care unit (ICU). The patient received an immediate transfusion of one unit of platelet. He also received fluid resuscitation for hypotension and high lactic acid (8 mmol/L). Meanwhile, norepinephrine (0.3 µg/kg/min) was used to maintain his blood pressure. He had no oliguria. Peripheral blood tests upon ICU admission showed highly elevated WBC of 23.55 × 10E^9^/L, Hb level of 85 g/L, hematocrit of 24.5%, and platelet count of 42 × 10E^9^/L. Urinalysis was normal. Biochemical tests showed elevated liver enzymes with peak AST of 3320 IU/L, ALT of 1780 IU/L. The prothrombin time (PT) was 13.7 seconds, the activated partial thromboplastin time (APTT) was 49 (normal range 28‐40 at our hospital) seconds and the fibrinogen concentration (Fib) was 2.89 G/L. hypersensitive C‐reactive protein was 8.14 mg/L, and procalcitonin was 1.49 ng/mL. Brain natriuretic peptide (BNP) was 215.54 pg/ml. Hepatitis A, B, C, and E viruses, HIV, Syphilis, Cytomegalovirus, and Epstein‐Barr virus were all tested negative by serology. Dengue virus antigen, IgM and IgG, and RNA were also tested negative. Triglyceride was normal (1.75 mmol/L). Ferritin was 3021.7 ng/mL. Upon further communication with his family members, his son recalled that the patient had been bitten by a rat one month ago. Peripheral blood sample on ICU day 1 was sent for mNGS test and also sent to Guangzhou CDC for serological testing of leptospirosis and Hantavirus. On ICU day 3, the initial bone marrow biopsy showed scattered hemophagocytic cells. On ICU day 4, the mNGS performed by IngeniGen Biotechnology Inc using Illumina MiniSeq detected SEOV with 28 high‐confidence sequence reads and mapped to an SEOV reference genome (S segment: NC_005236.1, M segment: NC_005237.1, L segment: NC_005238.1) (Figure 3). The important laboratory results prior to admission and after ICU admission were summarized in Table 1. Serology of Hantavirus by enzyme‐linked immunosorbent assay (ELISA) was also positive for Hantavirus IgG, thus confirming the SEOV infection. Antibody titration test was not performed; therefore, the exact titer of the Hantavirus IgG was unknown. Blood, sputum, and urine cultures were all negative on ICU day 1. The sputum culture performed on ICU day 2 grew multi‐drug resistance (MDR) Acinetobacter baumannii. Cerebrospinal fluid culture and blood culture performed on ICU day 11 also grew MDR A. baumannii. He was treated with meropenemand and colistin for the bacterial infections. On ICU day 10, head CT indicated new intracranial hemorrhage (Figure 4). On ICU day 15, the patient suddenly developed shortness of breath, hypotension, tachycardia, and respiratory arrest, suggesting foramen magnum hernia. He received the mechanical ventilation. His CSF workup showed markedly increased red blood cells, suggesting an increased intracerebral hemorrhage. The patient died on ICU day 19.

**TABLE 1:** the laboratory data prior to admission and after ICU admission

	Prior to admission	After ICU admission	Normal range
WBC count	8.32	23.55	3.5‐9.5 × 10E^9^/L
platelet count	31	42	100‐350 × 10E^9^/L
hemoglobin	171	85	115‐150 g/L
hematocrit	52.5%	24.5%	40%‐50%
serum creatinine	83.4	Normal	31.8‐116 µmol/L
ALT	225	1780	3‐35 IU/L
AST	272	3320	13‐35 IU/L
PT	NA	13.7	11‐14.5 s
APTT	NA	49	28‐40 s
Fib	NA	2.89	2.0‐4.0 g/L
Dengue virus IgM	Weakly positive	Negative	Negative
Dengue virus IgG	Negative	Negative	Negative
Dengue virus antigen	Negative	Negative	Negative
RNA of dengue virus	NA	Negative	Negative
Hepatitis A,B, C, E	NA	Negative	Negative
HIV, Syphilis	NA	Negative	Negative
DNA copies of CMV	NA	<500 copies/mL	<500 copies/mL
DNA copies of EB	NA	<500 copies/mL	<500 copies/mL
Hantavirus IgG	NA	Positive	Negative
SEOV sequence reads	NA	28	0

Abbreviations: ALT, alanine aminotransferase; APTT, activated partial thromboplastin time; AST, aspartate aminotransferase; CMV, cytomegalovirus; EB, Epstein‐Barr virus; Fib, fibrinogen concentration; Hantavirus IgG, using ELISA methods; PT, prothrombin time; SEOV, Seoul virus; WBC, white blood cell.

## 3 DISCUSSION

The initial differential diagnosis in this case was very challenging, as our patient presented with intracranial hemorrhage but without the three common causes including head trauma, hypertension, and arterial malformations.^5^ His presentation of fever, thrombocytopenia, and transaminitis promoted us to investigate an infectious cause. However, the CT scan of his chest did not reveal typical signs of infection, and the patient did not have other typical presentations of infections such as headache, chills, abdominal pain, and diarrhea. Although the abdomen CT scan demonstrated renal damage with perirenal inflammation, he did not have abnormal urination and his urinalysis was normal. Because dengue fever is prevalent in Guangzhou, it was highly suspected initially, especially after the IgM was weakly positive, but the follow‐up serological and molecular tests for Dengue were all negative, suggesting the initial IgM was a false‐positive result. All other conventional microbiological tests were also negative. mNGS is a genre of technologies that use what is known as shotgun sequencing of clinical samples or pure microbial cultures in which random samples of analyte DNA or RNA are surveyed en masse, which can be used for meaningful clinical diagnostics in microbiology.^6^ Fortunately, mNGS was able to detect SEOV directly in the blood which led to a definitive diagnosis confirmed by the positive Hantavirus IgG test.

The typically clinical manifestation of SEOV infection is HFRS, characterized by fever, hemorrhage, and impaired kidney function. The HFRS is more prevalent in Northern and Central Eastern China, but relatively less common in Southern China. HFRS incidence rate in Guangdong was 0.01 to 0.49/100 000.^2^ The disease peaks in autumn and winter season,^2^ which is consistent with the timing of this case (October – November). Since our patient had a history of rat bite, even though the clinical presentation was atypical: no hemorrhagic manifestations of skin and mucosal membrane and no renal syndromes, the HFRS was suspected.

The extrarenal manifestations of HFRS are rare but have been reported previously. Park KH, et al reported extrarenal manifestations involving the major organs occurred in one‐third (23/73) of patients with HFRS during various stages, from the febrile phase to the diuretic phase.^7^ The spectrum of the extrarenal manifestations includes pancreatobiliary (8 cases), gastrointestinal (5 cases), cardiovascular (4 cases), central nervous system (3 cases), retroperitoneal (1 case), intramural (1 case), respiratory (1 case), compartment syndrome (1 case), and hemophagocytic lymphohistiocytosis (HLH) (1 case).^7^ Few case reports showed cerebral involvement associated with HFRS including cerebral hemorrhage,^8^ cerebral edema and small subarachnoid, epidural and focal brain stem hemorrhages,^9^ and brain infarction.^7^ Unfortunately, in our case, the hematoma tissue and cerebrospinal fluid were not saved for serological and molecular tests for further investigation, which is the major limitation of this study.

## 4 CONCLUSION

This report highlights the importance of suspecting SEOV infection in febrile patients with thrombocytopenia and elevated liver enzymes despite the absence of hemorrhagic manifestations of skin and mucosal membrane and renal syndromes. mNGS can be a powerful tool to detect SEOV directly in the blood. Intracranial hemorrhage and brain infarction as extrarenal manifestations of HFRS are rare. Clinicians should be aware of various extrarenal manifestations of SEOV infections.

## CONFLICT OF INTEREST

The authors declare no conflict of interest.
